# Genome-Wide Study of Structural Variants in Bovine Holstein, Montbéliarde and Normande Dairy Breeds

**DOI:** 10.1371/journal.pone.0135931

**Published:** 2015-08-28

**Authors:** Mekki Boussaha, Diane Esquerré, Johanna Barbieri, Anis Djari, Alain Pinton, Rabia Letaief, Gérald Salin, Frédéric Escudié, Alain Roulet, Sébastien Fritz, Franck Samson, Cécile Grohs, Maria Bernard, Christophe Klopp, Didier Boichard, Dominique Rocha

**Affiliations:** 1 INRA, UMR1313, Génétique Animale et Biologie Intégrative, Domaine de Vilvert, Jouy-en-Josas, France; 2 AgroParisTech, UMR1313, Génétique Animale et Biologie Intégrative, Domaine de Vilvert, Jouy-en-Josas, France; 3 INRA, UMR1388 Génétique, Physiologie et Systèmes d’Elevage, Castanet-Tolosan, France; 4 Université de Toulouse INPT ENSAT, UMR1388 Génétique, Physiologie et Systèmes d’Elevage, Castanet-Tolosan, France; 5 Université de Toulouse INPT ENVT, UMR1388 Génétique, Physiologie et Systèmes d’Elevage, Toulouse, France; 6 INRA, SIGENAE, UR 875, INRA Auzeville, BP 52627, Castanet-Tolosan, France; 7 Union Nationale des Coopératives Agricoles d’Elevage et d’Insémination Animale, Paris, France; 8 INRA, UR1077, Mathématique Informatique et Génome, Domaine de Vilvert, Jouy-en-Josas, France; Wageningen UR Livestock Research, NETHERLANDS

## Abstract

High-throughput sequencing technologies have offered in recent years new opportunities to study genome variations. These studies have mostly focused on single nucleotide polymorphisms, small insertions or deletions and on copy number variants. Other structural variants, such as large insertions or deletions, tandem duplications, translocations, and inversions are less well-studied, despite that some have an important impact on phenotypes. In the present study, we performed a large-scale survey of structural variants in cattle. We report the identification of 6,426 putative structural variants in cattle extracted from whole-genome sequence data of 62 bulls representing the three major French dairy breeds. These genomic variants affect DNA segments greater than 50 base pairs and correspond to deletions, inversions and tandem duplications. Out of these, we identified a total of 547 deletions and 410 tandem duplications which could potentially code for CNVs. Experimental validation was carried out on 331 structural variants using a novel high-throughput genotyping method. Out of these, 255 structural variants (77%) generated good quality genotypes and 191 (75%) of them were validated. Gene content analyses in structural variant regions revealed 941 large deletions removing completely one or several genes, including 10 single-copy genes. In addition, some of the structural variants are located within quantitative trait loci for dairy traits. This study is a pan-genome assessment of genomic variations in cattle and may provide a new glimpse into the bovine genome architecture. Our results may also help to study the effects of structural variants on gene expression and consequently their effect on certain phenotypes of interest.

## Background

Over the past decade, many studies have attempted cataloging the nature and pattern of genomic alterations in population (e.g.[1]). The advent of novel high-throughput sequencing technologies [2–6] with the ability to partially or completely re-sequence genomes, in a relatively cost-effective manner, has offered new opportunities to study large scale genomic variations. In addition to single nucleotide polymorphisms (SNPs) and small insertions or deletions (indels), several other studies have identified larger and more complex structural variants (SVs). Originally, SVs were considered as genomic alterations affecting DNA segments greater than 1,000 base pairs (1 kbp) in size [7]. However, with new advances in high-throughput sequencing technologies, the operational spectrum of SVs has widened to include much smaller genomic alteration events (> 50 bp in size) [8]. SVs such as large insertions, large deletions, inversions, duplications, translocations and Copy Number Variants (CNVs), are less frequent than SNPs and indels within a given genome however some of them may have more significant functional effects [9] and may also play a role in genome structure remodeling [10–16]. For example, during its pilot phase, the 1000 Genomes Project Consortium has sequenced 185 human whole-genomes and has identified more than 22,025 deletions and 6,000 additional SVs [17]. Some of those SVs are associated with disease susceptibility, such as autism [18–20] or schizophrenia [21–23] in humans.

Many animal genomes have now been sequenced, including the genomes of several bulls and cows [24–48]. For example, Eck et al. (2009) generated the first cattle genome sequence by a next-generation sequencing method [24]. By sequencing a Fleckvieh bull genome, they discovered more than 2 million novel cattle SNPs. More recently, Daetwyler et al. (2014) have sequenced the whole-genome of 234 bulls from four different breeds and have identified more than 28 million variants (SNPs and indels). These polymorphisms have then been used to identify putative causative mutations for genetic defects or economically important complex traits [44].

Studies of large genomic variations in cattle have mostly focused on CNVs [27,29,32,43,49–63]. Some of these alterations have been involved in important phenotypes, such as resistance or susceptibility to gastrointestinal nematodes in Angus cattle [64–66] or feed intake in Holstein cows [67]. Other studies have also reported the involvement of other types of structural variants such as deletions, duplications or translocations in inherited disorders or coat colour patterning [31,38,68–75]. More recently McDaneld et al. have found a 70 kb-long deletion on BTA5 associated with decreased female reproductive efficiency in Bos indicus [76]; while Kadri et al. found a 660-kb long deletion on BTA12 with antagonistic effects on female fertility and milk production in Nordic Red cattle [77].

Here, we performed a large scale study to investigate both small indels (< = 50 bp) and large SVs (> 50 bp) in cattle by sequencing the whole-genome of 62 bulls from the three French major dairy breeds (Holstein, Montbéliarde and Normande breeds).

The collection of SVs reported in this study may prove useful to study their potential effect on the expression levels of certain genes of interest and consequently to study their link with the genetic variability of economically important traits in cattle.

## Materials and Methods

### Animal ethics

No animal experimentation was used in this study, therefore no ethical permission was required from any relevant authority. Sequencing was performed using genomic DNA obtained from sperm collected from semen straws kindly provided by approved commercial artificial insemination stations as part of their regular semen collection process. The authors did not participate in the acquisition of semen samples for the purpose of this research.

### Genomic DNA extraction

Genomic DNAs were extracted from semen of 62 dairy bulls (27 Holstein, 17 Montbéliarde and 18 Normande bulls) chosen based on their genetic contribution to the French cattle populations, using the Wizard Genomic DNA Purification Kit (Promega, Charbonnières-les-Bains, France) or using a standard phenol-chloroform method, respectively. A quality control inspection of each purified DNA sample was performed by agarose gel electrophoresis. DNA concentration was then measured with a Nanodrop ND-100 instrument (Thermo Scientific, Ilkirch, France).

### Library construction and sequencing

Genomic libraries were prepared using the TruSeq DNA Sample Preparation Kit (Illumina) according to the manufacturer’s instructions. Briefly, 4 μg genomic DNA were fragmented into 150–400 bp pieces using divalent cations at 94°C for 8 min. The resulting cleaved DNA fragments were purified using Agencourt AMPure XP beads (Beckman Coulter, Villepinte, France), then subjected to end-repair and phosphorylation and subsequent purification was performed using Agencourt AMPure XP beads (Beckman Coulter). These repaired DNA fragments were 3′-adenylated producing DNA fragments with a single ‘A’ base overhang at their 3′-ends for subsequent adapter-ligation. Illumina adapters were ligated to the ends of these 3′-adenylated DNA fragments followed by two purification steps using Agencourt AMPure XP beads (Beckman Coulter). Ten rounds of PCR amplification were performed to enrich the adapter-modified DNA library using primers complementary to the ends of the adapters. The PCR products were purified using Agencourt AMPure XP beads (Beckman Coulter) and size-selected (200 ± 25 bp) on a 2% agarose Invitrogen E-Gel (Thermo Scientific). Libraries were then checked on an Agilent Technologies 2100 Bioanalyzer using the Agilent High Sensitivity DNA Kit and quantified by quantitative PCR with the QPCR NGS Library Quantification kit (Agilent Technologies, Massy, France). Libraries were used for 2×100 bp paired-end sequencing on an Illumina HiSeq2000 with a TruSeq SBS v3-HS Kit (Illumina).

### Alignment to the reference

Sequence alignments were carried out using the Burrows-Wheeler Alignment tool (BWA v0.6.1-r104) [78] with default parameters for mapping reads to the UMD3.1 bovine reference genome [79]. Potential PCR duplicates, which can adversely affect the variant calls, were removed using the MarkDuplicates tool from Picard version 1.4.0 [80]. Only properly paired reads with a mapping quality of at least 30 (−q = 30) were kept. The resulting BAM files were then used for all subsequent analysis.

### Identification of small insertions and deletions

Small indels were detected using the Genome Analysis Tool Kit 2.4–9 (GATK) version and GATK-UnifiedGenotyper as SNP caller [81]. Prior to variant discovery, reads were subjected to local realignment, coordinate sort, quality recalibration, and PCR duplicate removal. In the GATK analysis, we used a minimum confidence score threshold of Q30 with default parameters. We have also used multi-sample variant calling in order to distinguish between a homozygous reference genotype and a missing genotype in the analyzed samples.

### Identification of SVs

Bioinformatics detection of potential genomic variation events was carried out on the 62 BAM files. We have performed multi-sample variant calling by Pindel software, v. 0.2.4y [82] using parameters as described in https://trac.nbic.nl/pindel. We first set the "Maximum event size index" to 9 in order to detect events whose sizes are up to 8,286,208 bp. We also set the–m parameter (min_perfect_match_around_BP) to 30 (i.e. at the point where the read is split into two, there should at least be 30 perfectly matching bases between the read and the reference sequences). We required a minimum mapping quality of the split read of 30 to support a breakpoint or junction. We finally used a custom python script to filter out Pindel-generated raw data: Only samples presenting at least three unique reads at the breakpoint of SVs were declared positive for the corresponding SV.

### Annotation of SV regions

Analyses of the overlap between SVs and functional elements were performed based on the gene build 77 database for the UMD3.1 bovine gene dataset obtained from the Ensembl Genome Browser using the Biomart software (http://www.ensembl.org/index.html). Positions of SV breakpoints predicted by Pindel were compared to gene start and end positions in order to identify SVs that may encompass an entire gene, those that overlap with exons of a given gene, those that overlap gene starts or ends and those for which both SV breakpoints are located within two different genes.

The Ensembl Biomart software was also used to find gene paralogs located within or overlapping the annotated SV regions.

Gene Ontology (GO) enrichment was also performed using the MouseMine analysis tools, a powerful new system for accessing MGI (Mouse Genome Informatics) data, using the InterMine framework and is available at the MGI international database resources (http://www.mousemine.org/mousemine/begin.do).

In order to investigate QTL regions within SV regions, we first downloaded all Bovine QTL regions from the public cattle QTL database release 24 (Aug 25, 2014), available at http://www.animalgenome.org. QTLs linked to milk traits (fat and protein content and yield) and somatic cell scores were subsequently extracted. A custom python script was then used to search for SVs located within or overlapping with QTLs regions.

### SV validation by high-throughput genotyping

In order to investigate our approach efficiency to detect SVs, we developed a genotyping-based strategy using the already available Illumina BovineLD custom BeadChip [83]. With this strategy, many individuals can be genotyped for many SVs at limited cost. The main idea was to convert predicted SVs into “virtual SNPs” by testing the base change at the SV breakpoints. Therefore, several selection filters were applied in order to select a panel of SVs for validation: **(1)** in order to overcome genotyping problems due to sequence repeats, the SV flanking sequences were first analyzed with the RepeatMasker software [84] and all SVs with masked flanking sequences were removed; **(2)** For deletions, if the first nucleotide of the deleted region is different from the first nucleotide which is located immediately after the SV 3’ breakpoint, then we selected the corresponding SVs for further analysis. This deletion was then converted into a “virtual SNP” for which the reference allele corresponds to the first nucleotide of the deleted region and the alternative allele corresponds to the first nucleotide immediately after the SV 3’ breakpoint; **(3)** For inversion, if the first nucleotide of the SV region is different from the reverse-complement of the last nucleotide of the same SV region, then we selected the corresponding SV for further analysis. This inversion was then converted into a “virtual SNP” for which the reference allele corresponds to the first nucleotide of the inverted region and the alternative allele corresponds to the reverse-complement of the last nucleotide of the same inverted region. Steps 2 and 3 were repeated with the reverse-complement sequences.

After applying the above filters, 331 deletions and inversions were selected for validation. They were genotyped for a large number of animals. High-throughput genotyping reactions were performed at Labogena core facility, using the custom low-density Illumina BovineLD SNP chip (San Diego, CA). SNPs with an Illumina design score above 0.4 were retained for further analysis. Oligonucleotides were designed, synthesized, and used to genotype 382 animals from at least eight major dairy breeds (Table 1). Several other breeds such as beef breeds (Limousine: 15, Charolaise: 19, Blonde d’Aquitaine: 12, Parthenaise: 12 and Gasconne: 9) were also included in our genotyping panel. However, none of the bulls used for SV identification was included in this genotyping sample list.

**Table 1:** Breed distribution of animals used in the validation study.

Breed	number of animals
**Holstein**	29
**Montbeliarde**	32
**Normande**	30
**Abondance**	29
**Brown Swiss**	30
**Pie rouge des plaines**	9
**Simmental**	16
**Tarentaise**	27
**Others**	180
**Total**	**382**

Table 1 summarizes the sample panel that was used for genotyping assays.

### Analysis of population structure

To indirectly validate the results of this SV detection study, we compared the population structure assessed from SVs to those previously obtained with SNPs [85]. We first performed Principal Components Analysis (PCA) using “dudi.pca” implemented in the R package ade4 [86] using all validated SV information. Second, we used the STRUCTURE software package [87] to assess the population structure. This program implements a model-based clustering method to infer population structure using genotype data of unlinked markers. We used the admixture model and correlated allele frequency version of STRUCTURE [88].

## Results and Discussion

### Whole-genome sequencing, read mapping

Sixty-two of the most contributing bulls from the three major French dairy breeds (Holstein, Montbeliarde and Normande) were selected for whole-genome sequencing. A total of 31,140 million raw paired-end reads with a length of 100 bases were generated, resulting in a total of 3,114 gigabases. Each sample was sequenced on 1–4 lanes and approximately 140 to 1,120 million paired-ends reads were obtained for each library. On average, 93% (from 75% to 97%) of the paired-end reads were properly aligned on the UMD3.1 bovine reference genome (S1 Table). Similar read mapping rates were obtained in other bovine whole-genome sequencing studies. For example, Kawahara-Miki et al. (2011) found that 86% of the paired-end reads they generated while sequencing the genome of a Japanese Kuchinoshima-Ushi bull mapped uniquely onto the bovine genome [25]. The average genome-wide sequence coverage from the mapped reads ranged from 5× to 42× across the different genomes, with 52 samples sequenced at least at 10 fold average coverage.

### Identification of genomic variations

Search for small variations with GATK-UnifiedGenotyer software resulted in the identification of 2,021,215 indels (S2 Table). On average we found 873,372 +/- 47,845 indels per bull. With this approach based on GATK, the largest indel identified was 11 bp in length.

**Fig 1:**
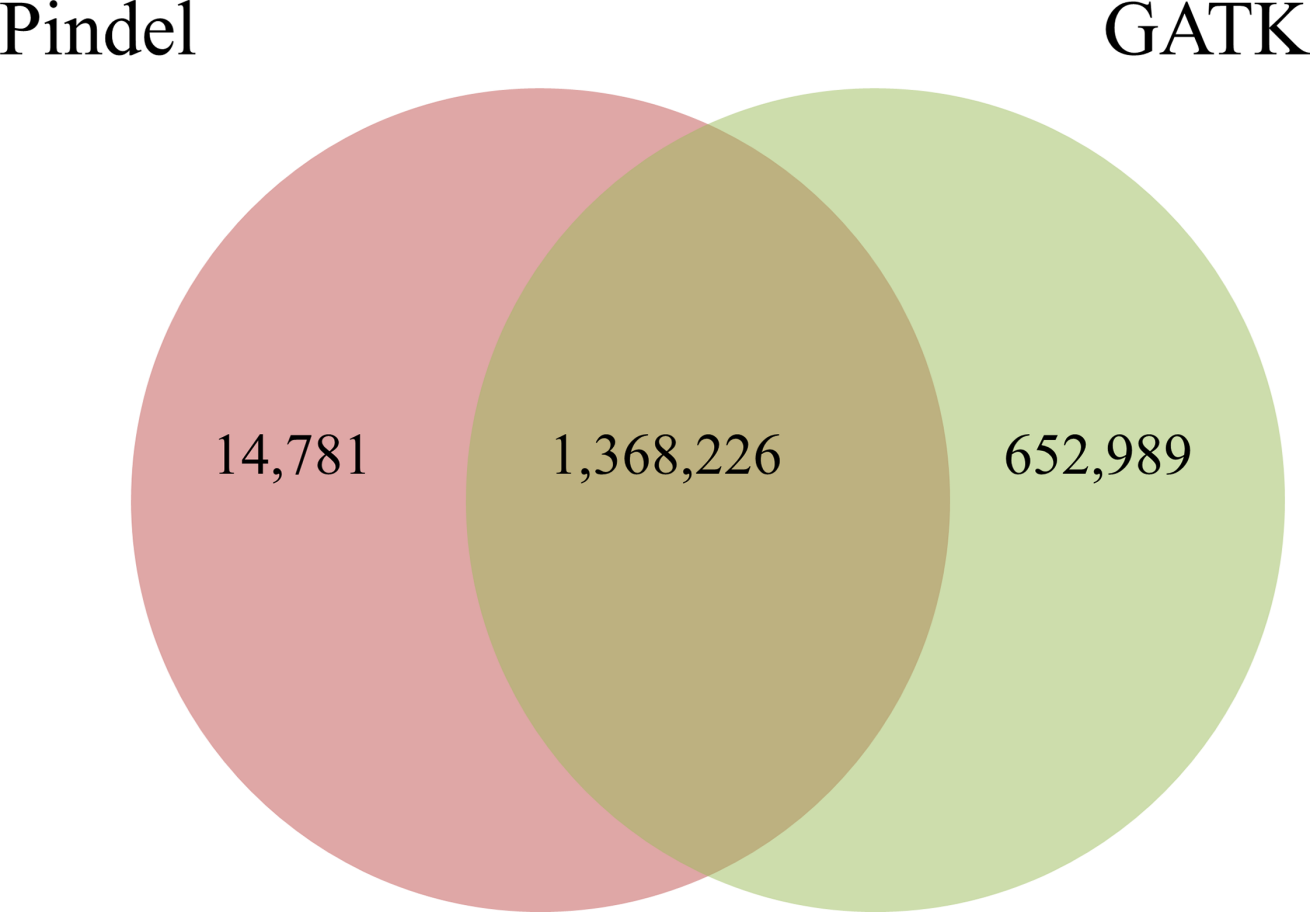
Small indels identified with GATK and Pindel. Venn diagram summarizing small indels identified by GATK and by Pindel.

With Pindel algorithm, we generated two categories of variations. First, we produced a catalog containing 1,384,490 small SVs mainly small insertions and deletions (< = 50 bp) out of which 1,383,007 small SVs were less than 11 bp in size (S2 Table). These were subsequently used for concordance analysis with small indels data generated by GATK. Almost 98.9% (1,368,226 out 1,383,007) of small indels detected by Pindel were also identified with GATK (Fig 1). This relatively high percentage of concordance suggests that most small SVs detected by Pindel might be true variations. However, it is difficult to precisely estimate the sensitivity (false-positive rate) of our SV detection method as small indels found with GATK but not with Pindel might not be true indels.

Second, we produced another catalog containing 6,426 putative large SVs (>50 bp) corresponding to 3,138 large deletions, 1,061 tandem duplications and 2,227 inversions (S3 Table). On average we observed nearly 199,200 small SVs and 305 large SVs per individual.

**Fig 2:**
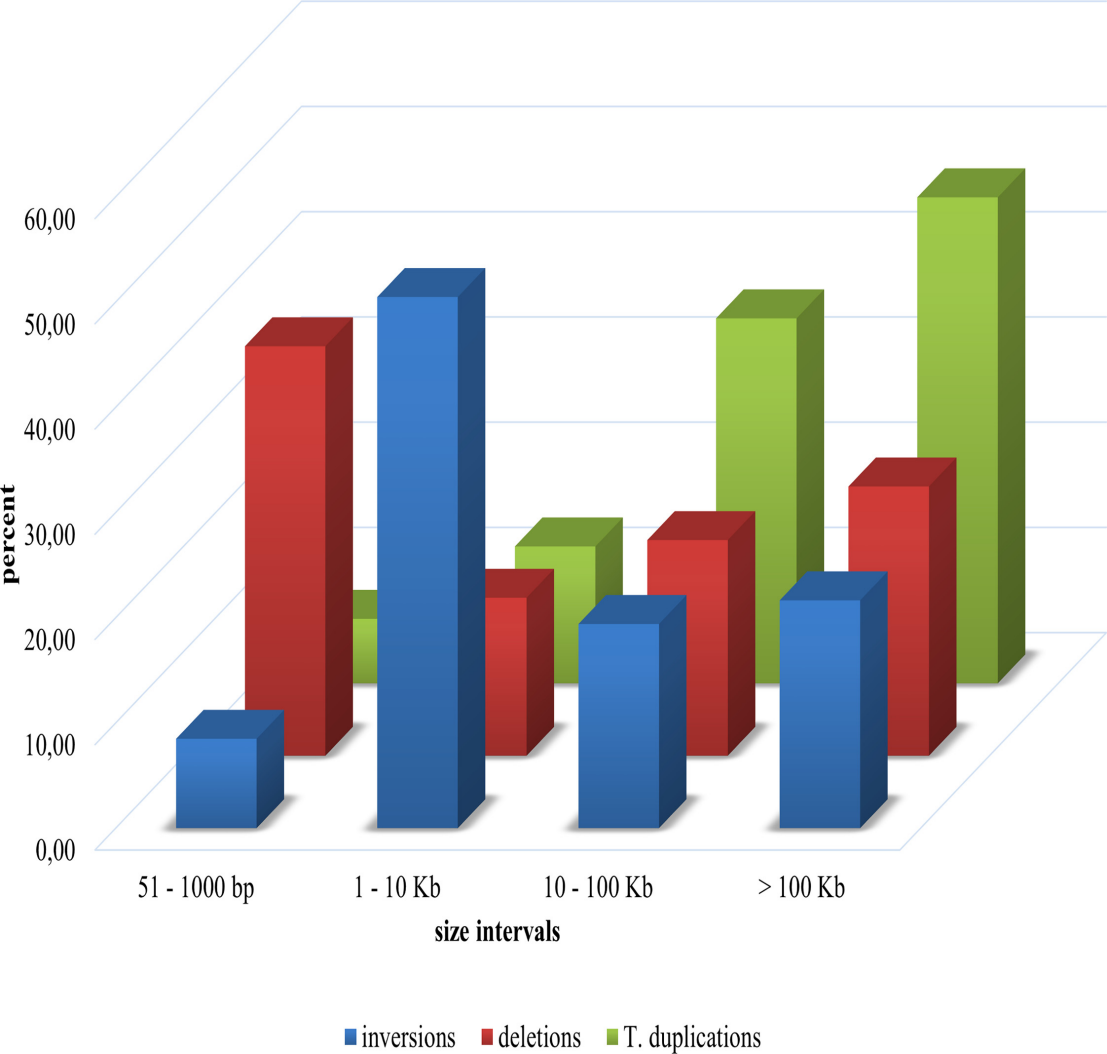
Distribution of SVs based on their type and size. Histogram summarizing the distribution of SVs based on their type and size. Inversions are highlighted in blue, deletions in red and tandem duplications in green.

Analysis of the length distribution of large SVs (Fig 2) revealed that most deletions (38.9%) are between 51 and 1,000 base pairs-long, whereas the length of most inversions (50.5%) is between 1 and 10 Kb while the vast majority of tandem duplications (80.9%) are larger than 10 Kb. These preliminary results seem to indicate a possible correlation between SV type and size. However, these observations should further be investigated.

**Fig 3:**
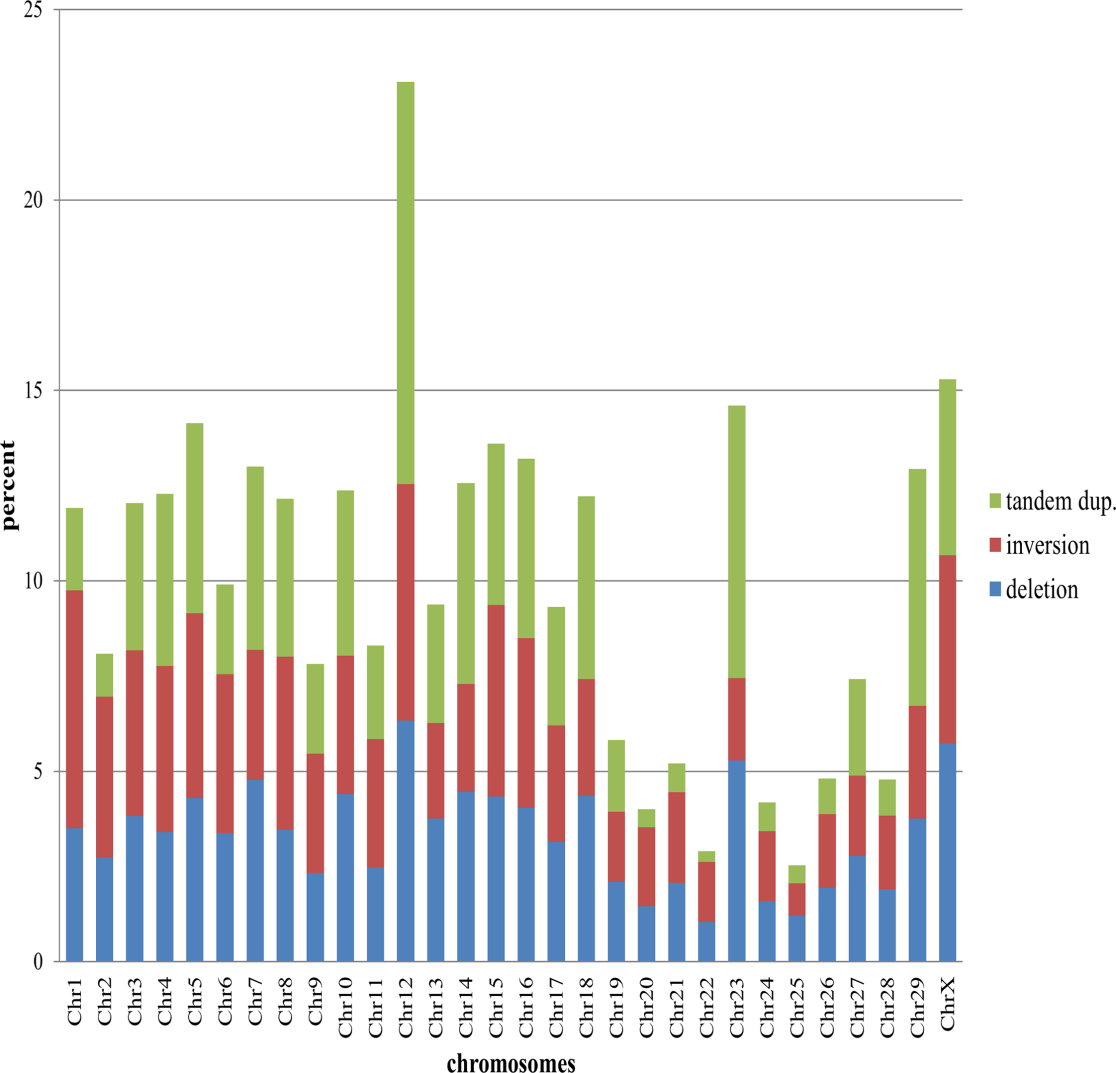
Chromosomal distribution of large SVs. Histogram showing the distribution of SVs within bovine chromosomes. Deletions are shown in blue, inversions in red and tandem duplications in green.

Analysis of the chromosomal distribution of the large SVs did not reveal any correlation with chromosome size (Fig 3). BTA12 harbours the highest number of SVs with approximately 7% of the total, followed by BTAX (5.5%) and BTA23 (5%). Moreover, no correlation has been observed between SV types and chromosomal distributions (Fig 3). The highest percentages of deletions were observed in BTA12 (6.3%), BTAX (5.7%) and BTA23 (5.3%). For tandem duplications, the highest percentages were observed in BTA1 (6.2%), BTA12 (6.2%) and BTA15 (5%). Finally, the highest percentages of inversions were observed in BTA12 (10.6%), BTA23 (7.2%) and BTA2 (6.2%).

Deletions and tandem duplications identified in this study covered a total length of up to 277 Mb corresponding to almost 10% of the whole bovine genome, whereas inversions covered a total length up to 152 Mb, ie almost 6% of the bovine genome. However, these percentages could be overestimated as SVs identified in our study are indeed putative variations and at this stage we do not know yet the false positive rate of our detection approach.

### Distribution of SVs between animals and between breeds

**Fig 4:**
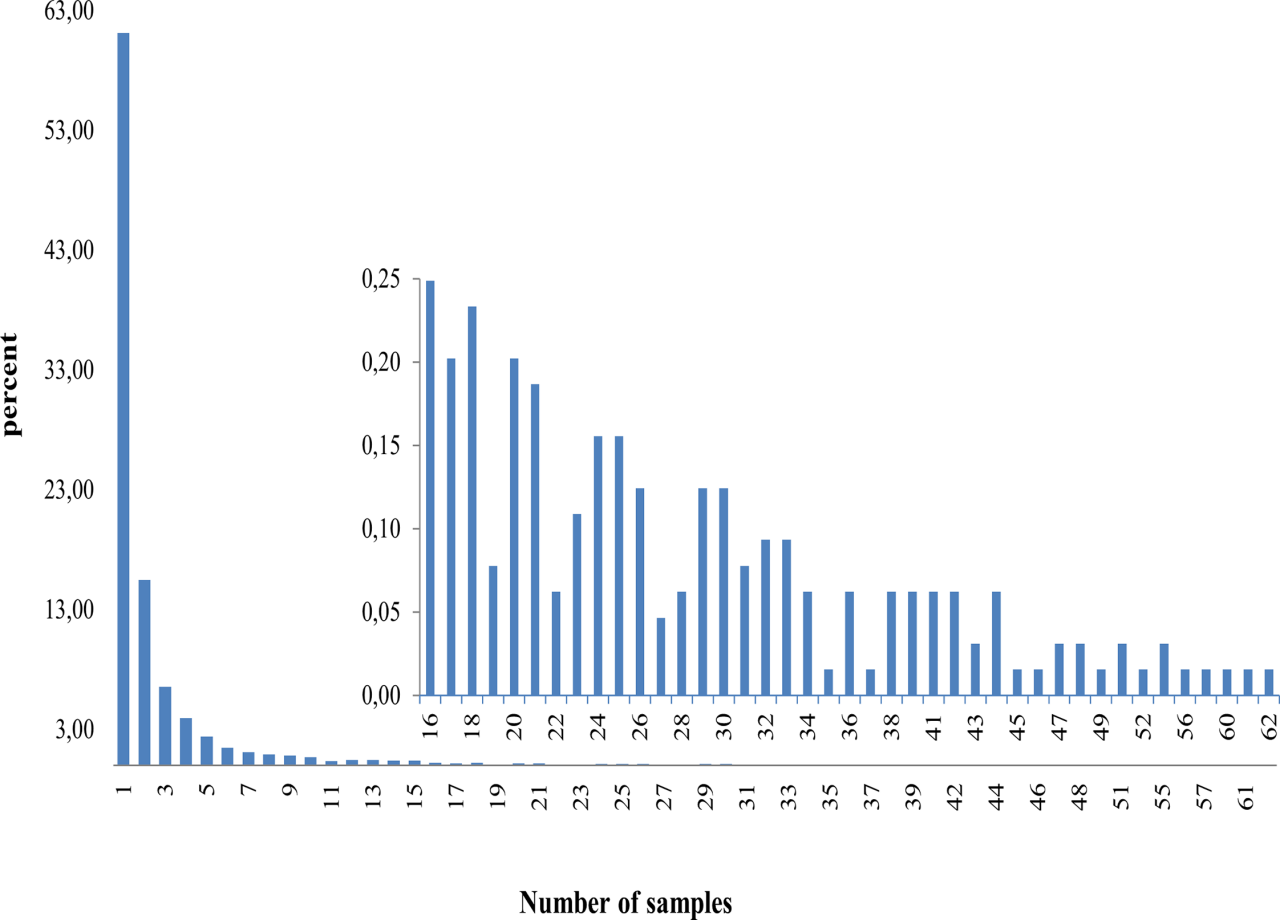
SV distribution among the 62 sequenced animals. Histogram showing the distribution of SVs among all 62 sequenced animals. Frequencies of SVs present in more than 16 sequenced samples were too low to be visualized and were therefore drawn in a separate graph embedded in the first one.

Overall, 61% of SVs were found only in single bulls (Fig 4). One deletion was found to be present in all 62 animals. One deletion and one tandem duplication were observed in 60 and 61 animals, respectively. Analysis of raw results generated by Pindel revealed that these 2 SVs were present in all 62 animals of our study but, for two animals, they were supported by less than the minimum number of 3 reads which was required to support an SV. These samples were therefore excluded from the final list of animals presenting the SVs. The cow genome reference sequence is derived from a single Hereford animal called Dominette. Therefore the first deletion and probably the two other SVs might be Hereford- or Dominette-specific SVs. Alternatively, these SVs could also be due to local errors in the UMD3.1 reference genome assembly.

**Fig 5:**
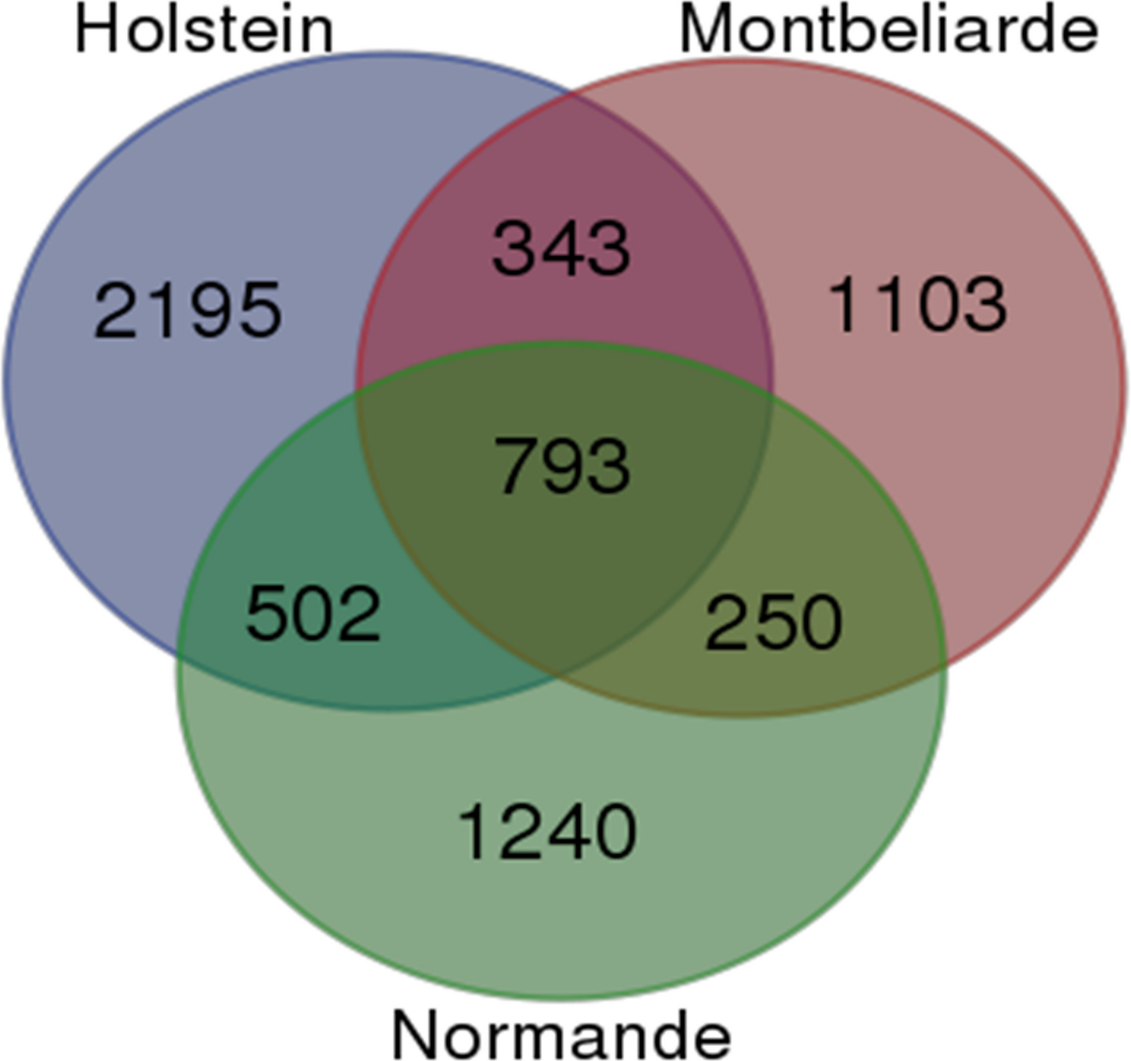
Distribution of SVs found within the three breeds. Venn diagram showing shared and unique SVs between the 3 breeds.

Comparison of large SVs revealed that 12% of these were shared between the three breeds (Fig 5) and at least one third were shared between at least two breeds. As shown in Fig 5, we identified more large SVs (2,195) in Holstein bulls than in Montbéliarde and Normande bulls (1,103 and 1,240, respectively). This result could be partly explained by the larger number of sequenced bulls in Holstein (27) than in Montbéliarde and Normande (18 and 17, respectively). Our results suggest that at least one third of the SV events occurred before the separation of the three breeds and therefore might also be present in other cattle breeds.

### Identification of potential CNV regions

CNVs are defined as loss (deletions) or gain (duplications) of copies of DNA segments. In order to identify SV regions (SVRs) that might correspond to potential CNV regions (CNVRs), we searched for DNA segments for which we could observe at the same time either a deletion in one bull and a duplication in another bull (across animals) or a deletion and a duplication at the same region within the same bull (within animal). We considered a given DNA region as a potential CNVR when the deleted and the duplicated segments are located within the same region, and are at least 70 percent overlapping.

In our study, we found 452 unique deletions and 392 unique duplications which may code for potential CNVRs (S4 Table).

In parallel, all deletion and tandem duplication regions identified in our study were also compared to publicly available CNVRs. Overall, 175 regions (128 deletion and 47 tandem duplication regions) overlapped with publicly available CNV datasets [29][43][52][56][60][65][89]. Out of these, 33 deletions and 29 tandem duplications were also identified with our first approach (S5 Table).

Overall, we identified 957 SVs that could potentially code for CNVs. Out of these, 547 SVs were deletions and 410 were tandem duplications (S4 and S5 Tables).

### Annotation of SVRs

#### Gene content

Analyses of functional elements lying within SVRs revealed a total of 2,415 (38%) SVRs which contain either entire gene-coding regions or only parts of genes (S6 Table). Therefore these SVs could potentially have an effect on expression of some of these genes and consequently a potential effect on some phenotypes. Out of these, 48% (1,168) were deletions, 27% (650) were tandem duplications and 25% (597) were inversions. Overall, a total of 5,011 genes overlap with these SVRs. The vast majority of these genes has paralogs (S7 Table) and correspond to uncharacterized genes (587) and genes coding for the olfactory receptor (327), U6 splicesomal RNA (159) and for the 5S ribosomal RNA (86).

Interestingly, we found 182 large deletions removing an entire gene. Overall, 115 different genes are affected by these large deletions (S8 Table). Almost 91.3% (105/115 genes) of these genes belong to large multigene families. The remaining large deletions remove 10 single-copy genes, out of which we found 3 pseudogenes, 3 protein coding genes and 4 genes encoding for microRNAs.

Alignment to the UMD3.1 bovine genome sequence of the sequence of the genes encoding for the novel miRNA ENSBTAG00000044935 and for bta-mir-2887-2 revealed several significant perfect matches (S9 Table), suggesting that multiple paralogous copies of these two microRNAs are located throughout the bovine genome.

A single perfect alignment match was however observed for the other two miRNAs. The gene encoding for bta-mir-2310 has been discovered in the normal adult bovine kidney (MDBK) cell line after infection with bovine herpesvirus 1 and shows a low expression in non- and infected cells [90]. Further analysis using TargetScan database [91] identified four genes to be targets of bta-mir-2310. These encode for interleukin 5, protein inhibitor of activated STAT 1 (PIAS1), solute carrier family 25 member 31 (SLC25A31) and zinc finger protein 316 (ZFN316). They are involved in different functions such as immune response, gene signaling, metabolite transport, and gene expression regulation. It is therefore possible that bta-mir-2310 plays an important role by negatively modulating the gene expression of these genes. However, its inactivation might also have limited impact as targets for numerous other miRNAs were also found in the 3’-untranslated regions of these four target genes.

The gene deleted by INRA_BovSV6339 encode for the mediator complex subunit 10 protein-coding gene (MED10) which is is a coactivator for DNA-binding factors that activate transcription of RNA polymerase II-dependent genes [92].

The other two genes deleted by INRA_BovSV1327 and by INRA_BovSV4164 encode for two yet uncharacterized proteins. The first gene contains only one exon and the predicted protein is around 100 amino acids. Alignment of this protein sequences against protein databases revealed a perfect match (100% identity) with the 3’-end of Bos taurus partitioning defective 3 homolog B isoform X5 (PARD3B). The second gene, however, contains 13 exons and code for a 463 amino acid protein. Amino acid sequence alignments against protein databases revealed high similarities with Bos Taurus ankyrin repeat domain-containing protein 26-like isoform X1 (LOC513969).

Further analyses are needed to check whether these deletions have any functional impact in cattle.

#### Gene Ontology

Gene Ontology analyses were also performed for all 5,011 genes and GO terms were obtained for biological processes, cellular components and molecular functions (S10 Table). Several GO terms were found to be significantly over-represented. For example, the five most enriched GO categories corresponding to biological process are related to metabolic process, primary metabolic process, organic substance metabolic process, single-organism metabolic process and cellular metabolic process.

#### QTLs in SVRs

The positions of the 6,426 predicted large SV events were also compared to the positions on the UMD3.1 bovine genome assembly of known quantitative trait loci (QTLs) deposited in the public database AnimalQTLdb [93]. Overall 587 SVs (246 large deletions, 236 inversions and 105 tandem duplications) were found located within or overlapping QTLs linked to milk traits and somatic cell count and scores (S11 Table). The most frequent traits corresponded to somatic cell score (257 SVs) followed by milk fat percentage (161 SVs), milk protein yield (143) and milk protein percentage (107 SVs). QTL enrichment analysis (S11 Table) showed no significant enrichment of specific QTLs linked to milk trait or somatic cell counts when comparing the SVs overlapping the QTL regions against SVs overlapping all other known QTL regions available in the AnimalQTLdb database for cattle.

### Validation of large SVs by genotyping

The efficiency of the selection approach and the relevance of the resulting SVs were assessed by genotyping a selected panel of SVs in 382 animals. None of the sequenced individuals was present in this genotyped panel.

Assays were developed for 331 putative SVs (S12 Table), out of which 255 (77%) were successfully genotyped (S13 Table) while genotyping failed for 76 (23%). These did not either cluster well according to genotype or failed to amplify most probably because of the sequence complexity or the presence of polymorphisms within flanking sequences or failed manufacture with Illumina. These were considered "failed assays". Out of the 255 successfully genotyped SVs, 237 were deletions and 18 were inversions.

For almost 25% (64 SVs) of the successfully genotyped SVs, only one SV allele was identified in all individuals (S13 and S14 Tables). Out of these, 61 SVs were homozygous for the reference allele and could therefore be incorrectly identified as true SVs by Pindel. Some of these SVs may also correspond to rare variants that were not present in the samples genotyped in this study. Indeed, almost 50% (30 out of 61) of these monomorphic SVs were found in a single bull and 84% (51 out of 61) were present in less than 5 animals.

The remaining 3 SVs were homozygous for the alternative allele and were therefore considered as true SVs.

Finally, 75% (191) of the successfully genotyped SVs were polymorphic and reliably scored, and thus were considered as true SVs (S13 and S14 Tables). Out of these, 184 SVs were deletions and 7 were inversions. The observed minor allele frequency (MAF) mean among true SVs was 0.20 ± 0.15 (SD), while the observed heterozygosity mean across loci was 0.28 ± 0.17, and the PIC (Polymorphic Information Content) mean was 0.23 ± 0.13 (S15 Table). Based on the observed heterozygosity and PIC rates in the validated SV panel and across the eight main breeds analyzed (S15 Table), we could conclude that this type of markers may be informative and is therefore of particular interest for linkage analysis.

Nine deletions overlapping with publicly available CNVs and 37 others identified as potential CNVs with our first approach were also validated in our genotyping study (S4 and S5 Tables).

### Assessment of population structure using SV genotyping data

Our validation study was carried out using animals from at least eight major dairy breeds (Table 1), out of which there were 29 Holstein, 32 Montbeliarde and 30 Normande animals.

**Fig 6:**
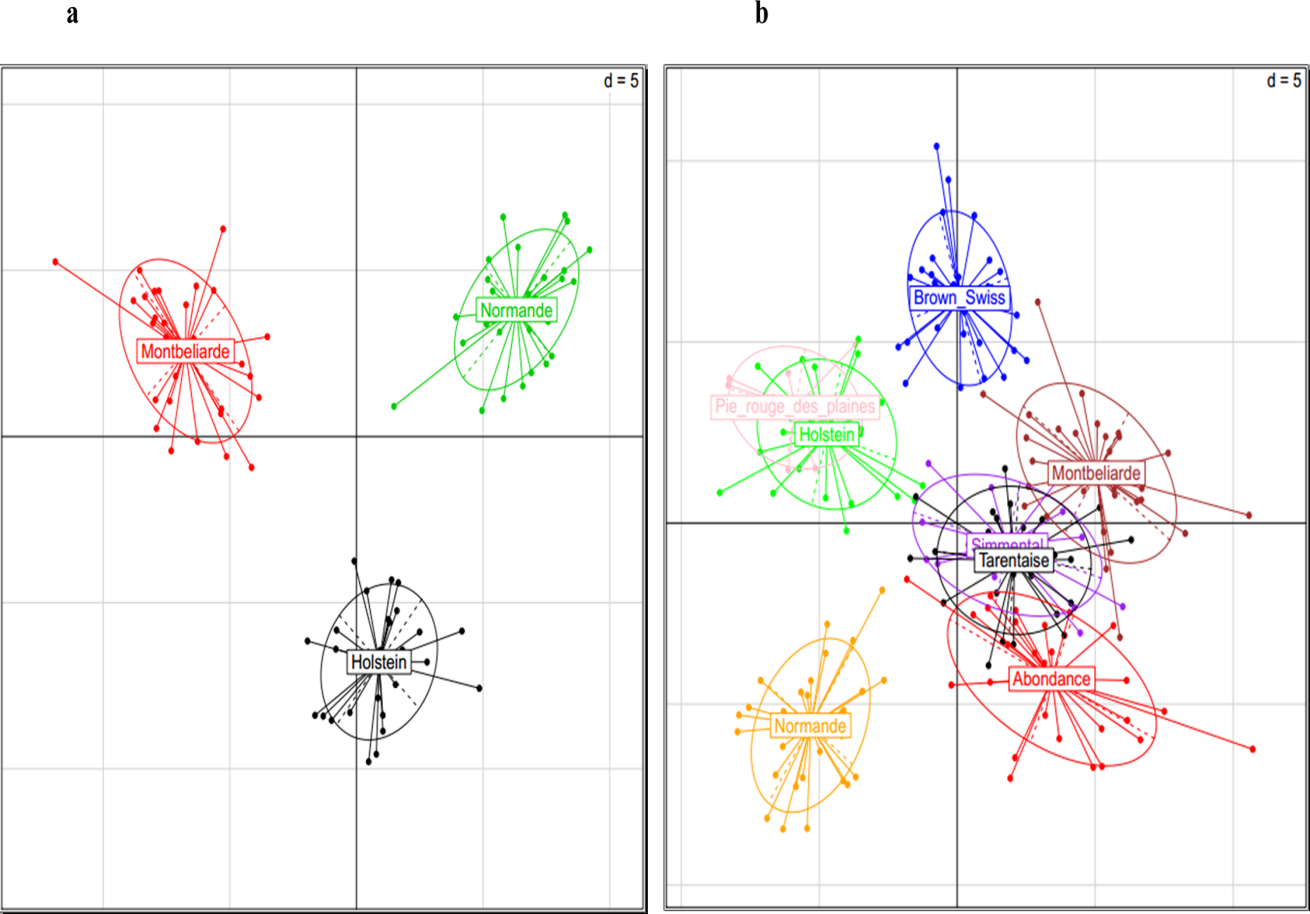
Results of PCA analysis. PCA analysis results were shown for the 3 main dairy breeds (Fig 6A) and for the 8 breeds (Fig 6B).

Using only genotyping data related to the three dairy breeds (S14 Table), PCA grouped individuals into three clusters according to their breeds of origin (Fig 6A).

**Fig 7:**
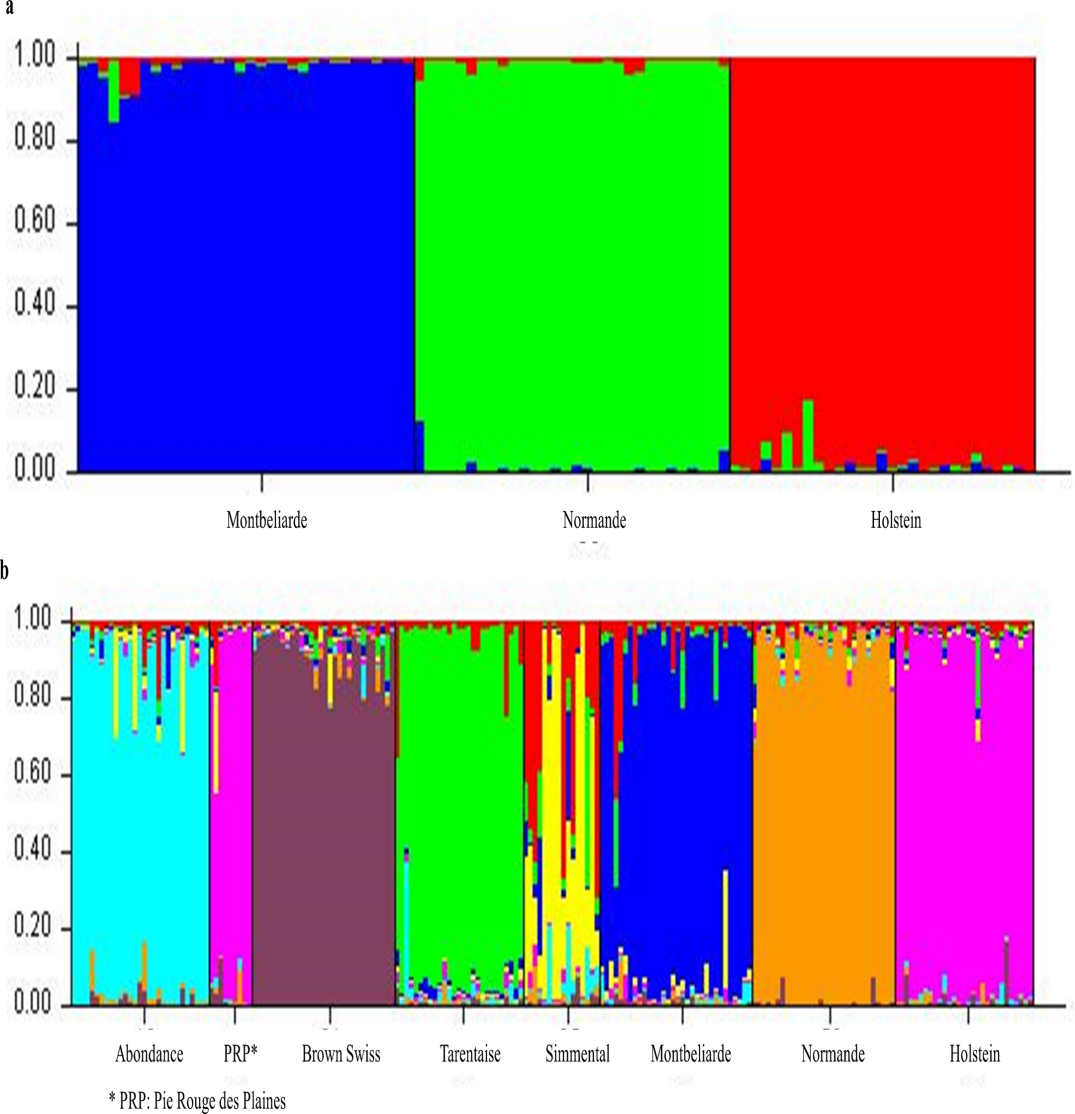
Genetic population structure prediction. Genetic population structure predicted by STRUCTURE software for the 3 main dairy breeds (Fig 7A) and for the 8 breeds (Fig 7B).

For K = 3, which corresponded to the three main breeds, STRUCTURE successfully sorted individuals into three groups entirely corresponding to the three breeds (Fig 7A).

Similar results were also observed with the eight breeds used in our validation study (Fig 6B and Fig 7B).

Our results are of particular interest as they could be considered as a global statistical validation step in addition to the genotyping validation approach we developed. Indeed, the SV used in these analyses provided a good description of the breed structure, similar to the one previously provided by SNP data [85].

## Conclusions

In the present study, we performed a pan-genome assessment of structural variations in cattle using whole genome sequence data. Analysis of WGS data of 62 bulls from the three main dairy breeds used in France (Holstein, Montbéliarde and Normande breeds) allowed the identification of 6,426 large SVs (> 50 bp). Out of these, 547 deletions and 410 tandem duplications were identified as potential CNVs.

To analyze the accuracy of our SV detection approach, a set of 331 SVs were selected for validation using a novel high-throughput genotyping strategy. Almost 75% of the successfully genotyped SVs could be validated and were polymorphic.

The collection of newly discovered SVs may prove useful to study their link with genetic variability of economically-important traits in cattle. It will be particularly interesting to analyze the impact of the large deletions inactivating completely single-copy genes.

## Supporting Information

S1 Table Reads mapping statistics. Summary of read mapping of the 62 bovine whole-genomes.
(XLSX)

S2 Table Small indels. Summary of all variants identified by GATK and by Pindel in all 62 samples.
(XLSX)

S3 Table Large SV. List of all 6,426 putative large SVs identified by Pindel.
(XLSX)

S4 Table List of overlapping deletions and tandem duplications. Overlapping deletions and duplications were considered as potential CNVs.
(XLSX)

S5 Table List of SVs that overlap with publicly available CNVs. Results of comparison between deletions and tandem duplication regions identified in this study with publicly available CNV regions
(XLSX)

S6 Table Gene content in SVs. List of genes located within or overlapped with SV regions.
(XLSX)

S7 Table Gene description and counts of genes and their paralogs. Counts of gene based on gene description and number of paralogs corresponding to each gene description.
(XLSX)

S8 Table Large deletions missing completely genes. List of deletions that remove completely a complete gene coding region. Deletions that remove completely a single copy gene (gene with no known paralog) were highlighted in bold and italics.
(XLSX)

S9 Table miRNA sequence homology search. Results of alignment of miRNA gene sequences onto the UMD3.1 genome.
(XLSX)

S10 Table GO enrichment. Results of gene ontology analysis for biological process, cellular component and molecular function enrichment.
(XLSX)

S11 Table SVs overlapping with QTLs. List of SV regions overlapping with publicly available QTL regions related to milk traits.
(XLSX)

S12 Table SVs used for genotyping. List of SVs present in the custom LD chip used for validation study.
(XLSX)

S13 Table Successfully genotyped SVs. List of SVs for which we obtained a good quality genotype.
(XLSX)

S14 Table Genotyping data. Individual genotype for all 191 validated SVs in the 8 major dairy breeds.
(XLSX)

S15 Table Frequencies of validated SVs. Estimation of allelic frequencies, Minor Allele Frequency (MAF), Heterozygosity (He) estimated by He = 2pq and Polymorphic Information Content (PIC) estimated by PIC = He-2p^2^q^2^.
(XLSX)

## References

[1] Altshuler DM, Gibbs RA, Peltonen L, Dermitzakis E, Schaffner SF, Yu F, et al (2010) Integrating common and rare genetic variation in diverse human populations. Nature 467: 52–58. 10.1038/nature09298 20811451 PMC3173859

[2] Margulies M, Egholm M, Altman WE, Attiya S, Bader JS, Bemben LA, et al (2005) Genome sequencing in microfabricated high-density picolitre reactors. Nature 437: 376–380. 16056220 10.1038/nature03959 PMC1464427

[3] Bentley DR, Balasubramanian S, Swerdlow HP, Smith GP, Milton J, Brown CG, et al (2008) Accurate whole human genome sequencing using reversible terminator chemistry. Nature 456: 53–59. 10.1038/nature07517 18987734 PMC2581791

[4] McKernan KJ, Peckham HE, Costa GL, McLaughlin SF, Fu Y, Tsung EF, et al (2009) Sequence and structural variation in a human genome uncovered by short-read, massively parallel ligation sequencing using two-base encoding. Genome Res 19: 1527–1541. 10.1101/gr.091868.109 19546169 PMC2752135

[5] Harris TD, Buzby PR, Babcock H, Beer E, Bowers J, Braslavsky I, et al (2008) Single-molecule DNA sequencing of a viral genome. Science 320: 106–109. 10.1126/science.1150427 18388294

[6] Drmanac R, Sparks AB, Callow MJ, Halpern AL, Burns NL, Kermani BG, et al (2010) Human genome sequencing using unchained base reads on self-assembling DNA nanoarrays. Science 327: 78–81. 10.1126/science.1181498 19892942

[7] Feuk L, Carson AR and Scherer SW (2006) Structural variation in the human genome. Nat Rev Genet 7: 85–97. 16418744 10.1038/nrg1767

[8] Alkan C, Coe BP and Eichler EE (2011) Genome structural variation discovery and genotyping. Nat Rev Genet 12: 363–376. 10.1038/nrg2958 21358748 PMC4108431

[9] Feuk L, Marshall CR, Wintle RF and Scherer SW (2006) Structural variants: changing the landscape of chromosomes and design of disease studies. Hum Mol Genet 15 Spec No: R57–R66. 16651370 10.1093/hmg/ddl057

[10] Iafrate AJ, Feuk L, Rivera MN, Listewnik ML, Donahoe PK, Qi Y, et al (2004) Detection of large-scale variation in the human genome. Nat Genet 36: 949–951. 15286789 10.1038/ng1416

[11] Sebat J, Lakshmi B, Troge J, Alexander J, Young J, Lundin P, et al (2004) Large-scale copy number polymorphism in the human genome. Science 305: 525–528. 15273396 10.1126/science.1098918

[12] Sharp AJ, Locke DP, McGrath SD, Cheng Z, Bailey JA, Vallente RU, et al (2005) Segmental duplications and copy-number variation in the human genome. Am J Hum Genet 77: 78–88. 15918152 10.1086/431652 PMC1226196

[13] Tuzun E, Sharp AJ, Bailey JA, Kaul R, Morrison VA, Pertz LM, et al (2005) Fine-scale structural variation of the human genome. Nat Genet 37: 727–732. 15895083 10.1038/ng1562

[14] Conrad DF, Andrews TD, Carter NP, Hurles ME and Pritchard JK (2006) A high-resolution survey of deletion polymorphism in the human genome. Nat Genet 38: 75–81. 16327808 10.1038/ng1697

[15] Hinds DA, Kloek AP, Jen M, Chen X and Frazer KA (2006) Common deletions and SNPs are in linkage disequilibrium in the human genome. Nat Genet 38: 82–85. 16327809 10.1038/ng1695

[16] McCarroll SA, Hadnott TN, Perry GH, Sabeti PC, Zody MC, Barrett JC, et al (2006) Common deletion polymorphisms in the human genome. Nat Genet 38: 86–92. 16468122 10.1038/ng1696

[17] Mills RE, Walter K, Stewart C, Handsaker RE, Chen K, Alkan C, et al (2011) Mapping copy number variation by population-scale genome sequencing. Nature 470: 59–65. 10.1038/nature09708 21293372 PMC3077050

[18] Weiss LA, Shen Y, Korn JM, Arking DE, Miller DT, Fossdal R, et al (2008) Association between microdeletion and microduplication at 16p11.2 and autism. N Engl J Med 358: 667–675. 10.1056/NEJMoa075974 18184952

[19] Kumar RA, KaraMohamed S, Sudi J, Conrad DF, Brune C, Badner JA, et al (2008) Recurrent 16p11.2 microdeletions in autism. Hum Mol Genet 17: 628–638. 18156158 10.1093/hmg/ddm376

[20] Marshall CR, Noor A, Vincent JB, Lionel AC, Feuk L, Skaug J, et al (2008) Structural variation of chromosomes in autism spectrum disorder. Am J Hum Genet 82: 477–488. 10.1016/j.ajhg.2007.12.009 18252227 PMC2426913

[21] Stefansson H, Rujescu D, Cichon S, Pietiläinen OPH, Ingason A, Steinberg S, et al (2008) Large recurrent microdeletions associated with schizophrenia. Nature 455: 232–236. 10.1038/nature07229 18668039 PMC2687075

[22] International Schizophrenia Consortium (2008) Rare chromosomal deletions and duplications increase risk of schizophrenia. Nature 455: 237–241. 10.1038/nature07239 18668038 PMC3912847

[23] Walsh T, McClellan JM, McCarthy SE, Addington AM, Pierce SB, Cooper GM, et al (2008) Rare structural variants disrupt multiple genes in neurodevelopmental pathways in schizophrenia. Science 320: 539–543. 10.1126/science.1155174 18369103

[24] Eck SH, Benet-Pagès A, Flisikowski K, Meitinger T, Fries R, Strom TM. (2009) Whole genome sequencing of a single Bos taurus animal for single nucleotide polymorphism discovery. Genome Biol 10: R82 10.1186/gb-2009-10-8-r82 19660108 PMC2745763

[25] Kawahara-Miki R, Tsuda K, Shiwa Y, Arai-Kichise Y, Matsumoto T, Kanesaki Y, et al (2011) Whole-genome resequencing shows numerous genes with nonsynonymous SNPs in the Japanese native cattle Kuchinoshima-Ushi. BMC Genomics 12: 103 10.1186/1471-2164-12-103 21310019 PMC3048544

[26] Zhan B, Fadista J, Thomsen B, Hedegaard J, Panitz F, Bendixen C. (2011) Global assessment of genomic variation in cattle by genome resequencing and high-throughput genotyping. BMC Genomics 12: 557 10.1186/1471-2164-12-557 22082336 PMC3248099

[27] Stothard P, Choi J-W, Basu U, Sumner-Thomson JM, Meng Y, Liao X, et al (2011) Whole genome resequencing of black Angus and Holstein cattle for SNP and CNV discovery. BMC Genomics 12: 559 10.1186/1471-2164-12-559 22085807 PMC3229636

[28] Canavez FC, Luche DD, Stothard P, Leite KRM, Sousa-Canavez JM, Plastow G, et al (2012) Genome sequence and assembly of Bos indicus. J Hered 103: 342–348. 10.1093/jhered/esr153 22315242

[29] Bickhart DM, Hou Y, Schroeder SG, Alkan C, Cardone MF, Matukumalli LK, et al (2012) Copy number variation of individual cattle genomes using next-generation sequencing. Genome Res 22: 778–790. 10.1101/gr.133967.111 22300768 PMC3317159

[30] Larkin DM, Daetwyler HD, Hernandez AG, Wright CL, Hetrick LA, Boucek L, et al (2012) Whole-genome resequencing of two elite sires for the detection of haplotypes under selection in dairy cattle. Proc Natl Acad Sci U S A 109: 7693–7698. 10.1073/pnas.1114546109 22529356 PMC3356612

[31] Capitan A, Allais-Bonnet A, Pinton A, Marquant-Le Guienne B, Le Bourhis D, Grohs C, et al (2012) A 3.7 Mb deletion encompassing ZEB2 causes a novel polled and multisystemic syndrome in the progeny of a somatic mosaic bull. PLoS One 7: e49084 10.1371/journal.pone.0049084 23152852 PMC3494662

[32] Choi J-W, Lee K-T, Liao X, Stothard P, An H-S, Ahn S, et al (2013) Genome-wide copy number variation in Hanwoo, Black Angus, and Holstein cattle. Mamm Genome 24: 151–163. 10.1007/s00335-013-9449-z 23543395

[33] Tsuda K, Kawahara-Miki R, Sano S, Imai M, Noguchi T, Inayoshi Y, et al (2013) Abundant sequence divergence in the native Japanese cattle Mishima-Ushi (Bos taurus) detected using whole-genome sequencing. Genomics 102: 372–378. 10.1016/j.ygeno.2013.08.002 23938316

[34] Liao X, Peng F, Forni S, McLaren D, Plastow G, Stothard P. (2013) Whole genome sequencing of Gir cattle for identifying polymorphisms and loci under selection. Genome 56: 592–598. 10.1139/gen-2013-0082 24237340

[35] Jansen S, Aigner B, Pausch H, Wysocki M, Eck S, Benet-Pagès A, et al (2013) Assessment of the genomic variation in a cattle population by re-sequencing of key animals at low to medium coverage. BMC Genomics 14: 446 10.1186/1471-2164-14-446 23826801 PMC3716689

[36] Sonstegard TS, Cole JB, VanRaden PM, Van Tassell CP, Null DJ, Schroeder SG, et al (2013) Identification of a nonsense mutation in CWC15 associated with decreased reproductive efficiency in Jersey cattle. PLoS One 8: e54872 10.1371/journal.pone.0054872 23349982 PMC3551820

[37] McClure M, Kim E, Bickhart D, Null D, Cooper T, Cole J, et al (2013) Fine mapping for Weaver syndrome in Brown Swiss cattle and the identification of 41 concordant mutations across NRCAM, PNPLA8 and CTTNBP2. PLoS One 8: e59251 10.1371/journal.pone.0059251 23527149 PMC3603989

[38] Allais-Bonnet A, Grohs C, Medugorac I, Krebs S, Djari A, Graf A, et al (2013) Novel insights into the bovine polled phenotype and horn ontogenesis in Bovidae. PLoS One 8: e63512 10.1371/journal.pone.0063512 23717440 PMC3661542

[39] Fritz S, Capitan A, Djari A, Rodriguez SC, Barbat A, Baur A, et al (2013) Detection of haplotypes associated with prenatal death in dairy cattle and identification of deleterious mutations in GART, SHBG and SLC37A2. PLoS One 8: e65550 10.1371/journal.pone.0065550 23762392 PMC3676330

[40] Glatzer S, Merten NJ, Dierks C, Wöhlke A, Philipp U, Distl O. (2013) A Single Nucleotide Polymorphism within the Interferon Gamma Receptor 2 Gene Perfectly Coincides with Polledness in Holstein Cattle. PLoS One 8: e67992 23805331 10.1371/journal.pone.0067992 PMC3689702

[41] Koch CT, Bruggmann R, Tetens J and Drögemüller C (2013) A non-coding genomic duplication at the HMX1 locus is associated with crop ears in highland cattle. PLoS One 8: e77841 10.1371/journal.pone.0077841 24194898 PMC3806818

[42] Wiedemar N, Tetens J, Jagannathan V, Menoud A, Neuenschwander S, Bruggmann R, et al (2014) Independent polled mutations leading to complex gene expression differences in cattle. PLoS One 9: e93435 10.1371/journal.pone.0093435 24671182 PMC3966897

[43] Shin D-H, Lee H-J, Cho S, Kim HJ, Hwang JY, Lee CK, et al (2014) Deleted copy number variation of Hanwoo and Holstein using next generation sequencing at the population level. BMC Genomics 15: 240 10.1186/1471-2164-15-240 24673797 PMC4051123

[44] Daetwyler HD, Capitan A, Pausch H, Stothard P, van Binsbergen R, Brøndum RF, et al (2014) Whole-genome sequencing of 234 bulls facilitates mapping of monogenic and complex traits in cattle. Nat Genet 46: 858–865. 10.1038/ng.3034 25017103

[45] Kõks S, Reimann E, Lilleoja R, Lättekivi F, Salumets A, Reemann P, et al (2014) Sequencing and annotated analysis of full genome of Holstein breed bull. Mamm Genome 25: 363–373. 10.1007/s00335-014-9511-5 24770584

[46] Choi J-W, Liao X, Stothard P, Chung W-H, Jeon H-J, Miller SP, et al (2014) Whole-genome analyses of Korean native and Holstein cattle breeds by massively parallel sequencing. PLoS One 9: e101127 10.1371/journal.pone.0101127 24992012 PMC4081042

[47] Lee H-J, Kim J, Lee T, Son JK, Yoon H-B, Baek KS, et al (2014) Deciphering the genetic blueprint behind Holstein milk proteins and production. Genome Biol Evol 6: 1366–1374. 10.1093/gbe/evu102 24920005 PMC4079194

[48] Barris W, Harrison BE, McWilliam S, Bunch RJ, Goddard ME and Barendse W (2012) Next generation sequencing of African and Indicine cattle to identify single nucleotide polymorphisms. Anim Prod Sci 52: 133–142.

[49] Liu GE, Van Tassel CP, Sonstegard TS, Li RW, Alexander LJ, Keele JW, et al (2008) Detection of germline and somatic copy number variations in cattle. Dev Biol (Basel) 132: 231–237. 18817307 10.1159/000317165

[50] Liu GE, Ventura M, Cellamare A, Chen L, Cheng Z, Zhu B, et al (2009) Analysis of recent segmental duplications in the bovine genome. BMC Genomics 10: 571 10.1186/1471-2164-10-571 19951423 PMC2796684

[51] Matukumalli LK, Lawley CT, Schnabel RD, Taylor JF, Allan MF, Heaton MP, et al (2009) Development and characterization of a high density SNP genotyping assay for cattle. PLoS One 4: e5350 10.1371/journal.pone.0005350 19390634 PMC2669730

[52] Liu GE, Hou Y, Zhu B, Cardone MF, Jiang L, Cellamare A, et al (2010) Analysis of copy number variations among diverse cattle breeds. Genome Res 20: 693–703. 10.1101/gr.105403.110 20212021 PMC2860171

[53] Bae JS, Cheong HS, Kim LH, NamGung S, Park TJ, Chun JY, et al (2010) Identification of copy number variations and common deletion polymorphisms in cattle. BMC Genomics 11: 232 10.1186/1471-2164-11-232 20377913 PMC2859865

[54] Fadista J, Thomsen B, Holm L-E and Bendixen C (2010) Copy number variation in the bovine genome. BMC Genomics 11: 284 10.1186/1471-2164-11-284 20459598 PMC2902221

[55] Seroussi E, Glick G, Shirak A, Yakobson E, Weller JI, Ezra E, et al (2010) Analysis of copy loss and gain variations in Holstein cattle autosomes using BeadChip SNPs. BMC Genomics 11: 673 10.1186/1471-2164-11-673 21114805 PMC3091787

[56] Hou Y, Liu GE, Bickhart DM, Cardone MF, Wang K, Kim ES, et al (2011) Genomic characteristics of cattle copy number variations. BMC Genomics 12: 127 10.1186/1471-2164-12-127 21345189 PMC3053260

[57] Kijas JW, Barendse W, Barris W, Harrison B, McCulloch R, McWilliam S, et al (2011) Analysis of copy number variants in the cattle genome. Gene 482: 73–77. 10.1016/j.gene.2011.04.011 21620936

[58] Hou Y, Bickhart DM, Hvinden ML, Li C, Song J, Boichard DA, et al (2012) Fine mapping of copy number variations on two cattle genome assemblies using high density SNP array. BMC Genomics 13: 376 10.1186/1471-2164-13-376 22866901 PMC3583728

[59] Jiang L, Jiang J, Wang J, Ding X, Liu J and Zhang Q. (2012) Genome-wide identification of copy number variations in Chinese Holstein. PLoS One 7: e48732 10.1371/journal.pone.0048732 23144949 PMC3492429

[60] Jiang L, Jiang J, Yang J, Liu X, Wang J, Wang H, et al (2013) Genome-wide detection of copy number variations using high-density SNP genotyping platforms in Holsteins. BMC Genomics 14: 131 10.1186/1471-2164-14-131 23442346 PMC3639935

[61] Cicconardi F, Chillemi G, Tramontano A, Marchitelli C, Valentini A, Ajmone-Marsan P, et al (2013) Massive screening of copy number population-scale variation in Bos taurus genome. BMC Genomics 14: 124 10.1186/1471-2164-14-124 23442185 PMC3618309

[62] Zhang L, Jia S, Yang M, Xu Y, Li C, Sun J, et al (2014) Detection of copy number variations and their effects in Chinese bulls. BMC Genomics 15: 480 10.1186/1471-2164-15-480 24935859 PMC4073501

[63] Xu L, Cole JB, Bickhart DM, Hou Y, Song J, VanRaden PM, et al (2014) Genome wide CNV analysis reveals additional variants associated with milk production traits in Holsteins. BMC Genomics 15: 683 10.1186/1471-2164-15-683 25128478 PMC4152564

[64] Liu GE, Brown T, Hebert DA, Cardone MF, Hou Y, Choudhary RK, et al (2011) Initial analysis of copy number variations in cattle selected for resistance or susceptibility to intestinal nematodes. Mamm Genome 22: 111–121. 10.1007/s00335-010-9308-0 21125402

[65] Hou Y, Liu GE, Bickhart DM, Matukumalli LK, Li C, Song J, et al (2012) Genomic regions showing copy number variations associate with resistance or susceptibility to gastrointestinal nematodes in Angus cattle. Funct Integr Genomics 12: 81–92. 10.1007/s10142-011-0252-1 21928070

[66] Xu L, Hou Y, Bickhart DM, Song J, Van Tassell CP, Sonstegard TS, et al (2014) A genome-wide survey reveals a deletion polymorphism associated with resistance to gastrointestinal nematodes in Angus cattle. Funct Integr Genomics 14: 333–339. 10.1007/s10142-014-0371-6 24718732

[67] Hou Y, Bickhart DM, Chung H, Hutchison JL, Norman HD, Connor EE, et al (2012) Analysis of copy number variations in Holstein cows identify potential mechanisms contributing to differences in residual feed intake. Funct Integr Genomics 12: 717–723. 10.1007/s10142-012-0295-y 22991089

[68] Ohba Y, Kitagawa H, Kitoh K, Sasaki Y, Takami M, Shinkai Y, et al (2000) A deletion of the paracellin-1 gene is responsible for renal tubular dysplasia in cattle. Genomics 68: 229–236. 10995564 10.1006/geno.2000.6298

[69] Hirano T, Kobayashi N, Itoh T, Takasuga A, Nakamaru T, Hirotsune S, et al (2000) Null mutation of PCLN-1/Claudin-16 results in bovine chronic interstitial nephritis. Genome Res 10: 659–663. 10810088 10.1101/gr.10.5.659 PMC310863

[70] Drögemüller C, Distl O and Leeb T (2001) Partial deletion of the bovine ED1 gene causes anhidrotic ectodermal dysplasia in cattle. Genome Res 11: 1699–1705. 11591646 10.1101/gr.182501 PMC311120

[71] Sugimoto M, Furuoka H and Sugimoto Y (2003) Deletion of one of the duplicated Hsp70 genes causes hereditary myopathy of diaphragmatic muscles in Holstein-Friesian cattle. Anim Genet 34: 191–197. 12755819 10.1046/j.1365-2052.2003.00990.x

[72] Flisikowski K, Venhoranta H, Nowacka-Woszuk J, McKay SD, Flyckt A, Taponen J, et al (2010) A novel mutation in the maternally imprinted PEG3 domain results in a loss of MIMT1 expression and causes abortions and stillbirths in cattle (Bos taurus). PLoS One 5: e15116 10.1371/journal.pone.0015116 21152099 PMC2994898

[73] Meyers SN, McDaneld TG, Swist SL, Marron BM, Steffen DJ, O’Toole D, et al (2010) A deletion mutation in bovine SLC4A2 is associated with osteopetrosis in Red Angus cattle. BMC Genomics 11: 337 10.1186/1471-2164-11-337 20507629 PMC2891616

[74] Durkin K, Coppieters W, Drögemüller C, Ahariz N, Cambisano N, Druet T, et al (2012) Serial translocation by means of circular intermediates underlies colour sidedness in cattle. Nature 482: 81–84. 10.1038/nature10757 22297974

[75] Venhoranta H, Pausch H, Wysocki M, Szczerbal I, Hänninen R, Taponen J, et al (2013) Ectopic KIT copy number variation underlies impaired migration of primordial germ cells associated with gonadal hypoplasia in cattle (Bos taurus). PLoS One 8: e75659 10.1371/journal.pone.0075659 24086604 PMC3784456

[76] McDaneld TG, Kuehn LA, Thomas MG, Pollak EJ and Keele JW (2014) Deletion on chromosome 5 associated with decreased reproductive efficiency in female cattle. J Anim Sci 92: 1378–1384. 10.2527/jas.2013-6821 24492568

[77] Kadri NK, Sahana G, Charlier C, Iso-Touru T, Guldbrandtsen B, Karim L, et al (2014) A 660-Kb deletion with antagonistic effects on fertility and milk production segregates at high frequency in Nordic Red cattle: additional evidence for the common occurrence of balancing selection in livestock. PLoS Genet 10: e1004049 10.1371/journal.pgen.1004049 24391517 PMC3879169

[78] Li H and Durbin R (2009) Fast and accurate short read alignment with Burrows-Wheeler transform. Bioinformatics 25: 1754–1760. 10.1093/bioinformatics/btp324 19451168 PMC2705234

[79] Zimin A V, Delcher AL, Florea L, Kelley DR, Schatz MC, Puiu D, et al (2009) A whole-genome assembly of the domestic cow, Bos taurus. Genome Biol 10: R42 10.1186/gb-2009-10-4-r42 19393038 PMC2688933

[80] Picard Tools—By Broad Institute (n.d.). Available: http://broadinstitute.github.io/picard/.

[81] McKenna A, Hanna M, Banks E, Sivachenko A, Cibulskis K, Kernytsky A, et al (2010) The Genome Analysis Toolkit: a MapReduce framework for analyzing next-generation DNA sequencing data. Genome Res 20: 1297–1303. 10.1101/gr.107524.110 20644199 PMC2928508

[82] Ye K, Schulz MH, Long Q, Apweiler R and Ning Z (2009) Pindel: a pattern growth approach to detect break points of large deletions and medium sized insertions from paired-end short reads. Bioinformatics 25: 2865–2871. 10.1093/bioinformatics/btp394 19561018 PMC2781750

[83] Boichard D, Chung H, Dassonneville R, David X, Eggen A, Fritz S, et al (2012) Design of a bovine low-density SNP array optimized for imputation. PLoS One 7: e34130 10.1371/journal.pone.0034130 22470530 PMC3314603

[84] RepeatMasker Web Server (n.d.). Available: http://www.repeatmasker.org/cgi-bin/WEBRepeatMasker.

[85] Gautier M, Laloë D and Moazami-Goudarzi K (2010) Insights into the genetic history of French cattle from dense SNP data on 47 worldwide breeds. PLoS One 5: e13038 10.1371/journal.pone.0013038 20927341 PMC2948016

[86] Dray S and Dufour AB (2007) The ade4 package: implementing the duality diagram for ecologists. J Stat Softw 22: 1–20.

[87] Pritchard JK, Stephens M and Donnelly P (2000) Inference of population structure using multilocus genotype data. Genetics 155: 945–959. 10835412 10.1093/genetics/155.2.945 PMC1461096

[88] Falush D, Stephens M and Pritchard JK (2003) Inference of population structure using multilocus genotype data: linked loci and correlated allele frequencies. Genetics 164: 1567–1587. 12930761 10.1093/genetics/164.4.1567 PMC1462648

[89] Zhang Q, Ma Y, Wang X, Zhang Y and Zhao X (2014) Identification of copy number variations in Qinchuan cattle using BovineHD Genotyping Beadchip array. Mol Genet Genomics. 10.1007/s00438-014-0923-4 25248638

[90] Glazov EA, Kongsuwan K, Assavalapsakul W, Horwood PF, Mitter N and Mahony TJ. (2009) Repertoire of bovine miRNA and miRNA-like small regulatory RNAs expressed upon viral infection. PLoS One 4: e6349 10.1371/journal.pone.0006349 19633723 PMC2713767

[91] TargetScanHuman 6.2 (n.d.). Available: http://www.targetscan.org/.

[92] Sato S, Tomomori-Sato C, Banks CAS, Sorokina I, Parmely TJ, Kong SE, et al (2003) Identification of mammalian Mediator subunits with similarities to yeast Mediator subunits Srb5, Srb6, Med11, and Rox3. J Biol Chem 278: 15123–15127. 12584197 10.1074/jbc.C300054200

[93] Hu Z-L, Fritz ER and Reecy JM (2007) AnimalQTLdb: a livestock QTL database tool set for positional QTL information mining and beyond. Nucleic Acids Res 35: D604–D609. 17135205 10.1093/nar/gkl946 PMC1781224

